# Role of Single Photon Emission Computed Tomography in Epilepsy

**DOI:** 10.1155/2011/803920

**Published:** 2010-12-14

**Authors:** Sita Jayalakshmi, Pushpalatha Sudhakar, Manas Panigrahi

**Affiliations:** ^1^Department of Neurology, Krishna Institute of Medical Sciences, 1-8-31/1, Minister Road, Secunderabad, Andhra Pradesh 500 003, India; ^2^Department of Nuclear Medicine, Krishna Institute of Medical Sciences, Hyderabad, 1-8-31/1, Minister Road, Secunderabad, Andhra Pradesh 500 003, India; ^3^Department of Neurosurgery, Krishna Institute of Medical Sciences, Hyderabad, 1-8-31/1, Minister Road, Secunderabad, Andhra Pradesh 500 003, India

## Abstract

Molecular imaging with ictal single photon emission computed tomography (SPECT) is an established functional imaging modality for the presurgical evaluation of patients with refractory partial onset seizures. SPECT coregistered on to the MRI has greater sensitivity to identify the ictal onset zone. Ictal SPECT should always be interpreted in the context of other presurgical investigations. Ictal SPECT is sensitive method for the lateralization of TLE, but ictal SPECT is more sensitive when MRI is normal. Ictal SPECT and interictal PET are complementary to each other in lateralizing the side in patients with TLE and normal MRI. In extratemporal epilepsy, ictal SPECT will guide the placement of surface grid and depth electrodes.

## 1. Introduction

Molecular imaging with ictal and interictal single photon emission computed tomography (SPECT) is an established functional imaging modality for the presurgical evaluation of patients with refractory partial onset seizures. Ictal SPECT has the potential to localize the ictal onset zone noninvasively and accurately and provides complementary data during multimodality evaluation of the epileptogenic zone. Ictal SPECT is more sensitive than structural imaging [1] but gives little indication about the underlying pathology. SPECT co-registered on to the MRI has greater sensitivity to identify the ictal onset zone. It is usually assumed that the largest and most intense ictal hyperperfusion region is the ictal onset zone. Ictal SPECT injection should be performed in the video EEG unit by trained technicians. The dye should be injected fast [2, 3]. High resolution SPECT and MRI scanner are important with a need for good cooperation between neurology and nuclear medicine departments for a successful programme to be implemented.

## 2. Brain Perfusion Tracers

The brain perfusion tracers that cross the blood brain barrier with a long retention time in the brain are ^99m^technetium (^99m ^TC-labeled agents). The two commonly used tracers are ^99m^hexamethylene propylene amino (^99m^TC-HMPAO) and ^99m^TC-ethyl cysteinate dimer (^99m^TC-ECD). ^99m^TC-ECD is stable 6 to 8 hours and ^99m^TC-HMPAO for 4 hours. ^99m^TC-ECD is cleared from the body more rapidly than ^99m ^TC HMPAO and gives a higher brain to soft tissue activity ratio, and this improves the image quality [4] D.S.Lee et al. found that ^99m^TC-HMPAO ictal SPECT was superior to ^99m^TC-ECD ictal SPECT for localization of epileptogenic zone [5]. Early ictal injection is an important criterion for best results and is associated with high concordance with other studies [6]. S. K. Lee et al. reported that an injection delay of less than 20 seconds after seizure onset was significantly correlated with correct localization [7]. The SPECT images can be acquired up to 4 hours after this termination of the seizure.

### 2.1. Limiting Factors

Ictal SPECT has a poor time resolution. After injection of the tracer, it takes about 30s to reach the brain, and around 70% of the radioligand is taken up during first pass. An ictal perfusion SPECT image displays both ictal onset zone and seizure propagation pathways. The region with largest and most intense hyperperfusion is considered as the ictal onset zone. It has been shown that these regions may also represent ictal propagation [8]. Earlier ictal injection given during a seizure more likely suggests that the largest and most intense ictal perfusion area represents the ictal onset zone than the seizure propagation. Contralateral spread of ictal activity restricted to a region homotopic to the ictal onset zone results in a mirror image [9].

### 2.2. Interictal SPECT

The rationale for interictal SPECT imaging is to serve as a baseline reference study for the interpretation of ictal SPECT images. Interictal SPECT should be done after a seizure-free period of at least 24 hours, and the dye should be injected during EEG monitoring when there is no epileptiform activity.

### 2.3. Role of Ictal SPECT during Presurgical Evaluation of Refractory Partial Epilepsy

Ictal EEG is the gold standard investigation for the ictal onset zone. However scalp ictal EEG will not permit accurate localization in up to 40% patients with temporal lobe epilepsy if used alone. Concordance of ictal scalp EEG with MRI brain, ictal SPECT, and interictal PET improves the surgical outcome. As invasive EEG evaluation is associated with serious complications like intracerebral hemorrhage and infections, though rare, noninvasive modalities of presurgical evaluation strategies should always be performed to improve the accuracy of localization of epileptogenic zone.

The interpretation of ictal SPECT should always be done in the context of a full presurgical evaluation. The neurologist/epileptologist plays an important role in the interpretation of the SPECT images. The injection time should be known, as early injections give the best results.

### 2.4. SPECT in Temporal Lobe Epilepsy

**Figure 1:**
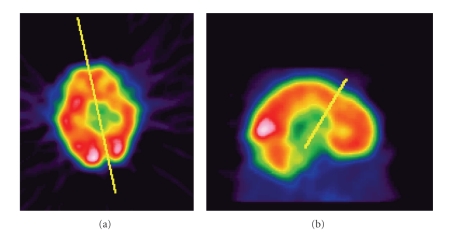
Reconstruction of temporal lobes (a) transaxial slices parallel to the temporal lobe with (b) coronal slices perpendicular to this plane.

The temporal lobes are best viewed by reconstructing transaxial slices parallel to the temporal lobe with coronal slices perpendicular to this plane [10–12]. The anterior-posterior commisure (AC-PC) line can be approximated by joining the bottom of the frontal lobe and occipital lobe on a midline sagittal slice, and temporal lobe plane is then derived from this line (Figure 1). Any asymmetry of more than 10% over the anterior temporal lobes during quantification is significant. Visual impression is a good indicator for interpretation, and quantification is usually only performed for research purposes [12, 13].

### 2.5. Interictal SPECT

Interictal SPECT has low sensitivity and accuracy in temporal lobe epilepsy when compared to FDGPET, as interictal blood flow changes are less marked than metabolic changes. In patients with temporal lobe epilepsy, interictal SPECT showed hypoperfusion in the side of epileptogenic focus in 55% and contralateral hypoperfusion leading to false lateralization in 10% [14]. The hypoperfusional most commonly involves the anterior pole of the temporal lobe and medial temporal region. The lateral temporal cortex and ipsilateral frontal and parietal cortex hypoperfusion may also be seen. The presence of left temporal lobar hypoperfusion has been shown to reduce the risk of a postoperative decline in verbal short-term memory function following left temporal lobectomy [15]. The present clinical role of interictal SPECT is to provide as a baseline for comparison and interpretation of ictal SPECT studies

### 2.6. Ictal SPECT

Ictal studies are obtained during the seizure. Postictal studies are obtained by injection after the completion of a seizure. The term peri-ictal SPECT refers to ictal and early postictal injections. Ictal SPECT is sensitive with correct identification of the seizure focus being achieved in more than 90%, and seizure-free outcome has been achieved in 60–80% of patients [11, 12, 16–25]. False lateralization has been reported in less than 5% of cases. The sensitivity of postictal SPECT injection was 70%, and false localization was reported in less than 5% of the cases [26].

### 2.7. Ictal SPECT Patterns

**Figure 2:**
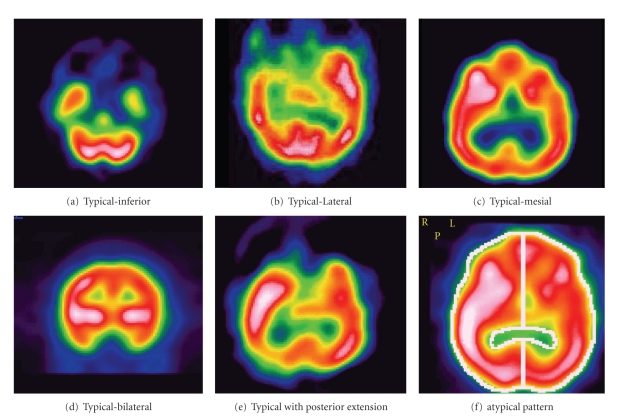
Ictal SPECT patterns in temporal epilepsy as described by Ho et al. [27].

The ictal SPECT hyperperfusion patterns were classified by Ho et al. into typical, typical with posterior extension, bilateral, and atypical patterns [27] (Figure 2). The outcome for seizure freedom at two years was 60%, 69%, 67% in the typical, typical with posterior extension, and bilateral pattern groups, suggesting that extended patterns of ictal perfusion represent seizure propagation pathways rather than intrinsically epileptogenic tissue. Atypical pattern group had a worse outcome with only 33% being seizure free and indicates diffuse or extra-temporal epileptogenicity. 

Temporal lobe hyperperfusion typically involves the anterior pole and medial temporal lobe with variable degree of involvement of the lateral temporal cortex. Hyperperfusion of the ipsilateral basal ganglia is common and correlates well with dystonic posturing of the contralateral arm during the seizure [28]. Hyperperfusion of ipsilateral thalamus may also be seen, but infrequent. Propagation of the seizure may lead to hyperperfusion of the contralateral medial temporal lobe, but it is less extensive and less in intensity than in the temporal lobe, where the ictal onset occurs [29]. Ipsilateral insula cortex and basal frontal lobe may also be involved. Ictal hyperperfusion is seen in TLE due to mesial temporal sclerosis and also with structural lesions [27].

The injected seizure type and ictal semiology should be known for a correct interpretation of ictal SPECT. The results are best during the injection of complex partial seizures while secondarily generalized seizures show hyperperfusion of multiple areas [30].

### 2.8. Ictal SPECT in TLE with Normal MRI

**Figure 3:**
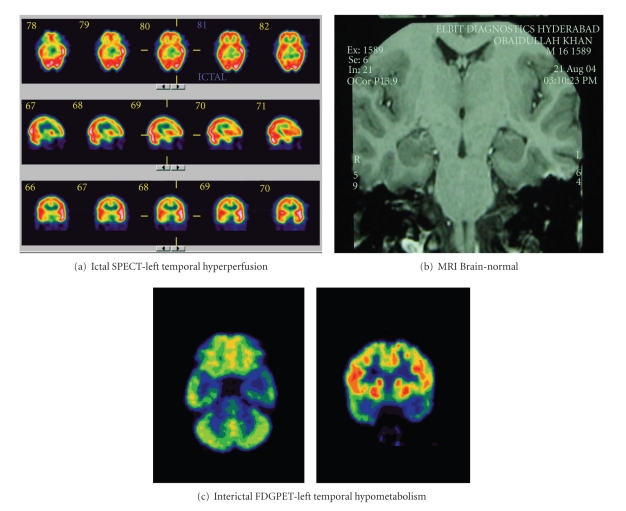
Ictal SPECT and FDGPET in a patient with refractory temporal lobe epilepsy and normal MRI showing lateralization to left. The patient underwent left temporal lobectomy with amygdalohippocampectomy and is seizure free for more than 3 years.

Patients with refractory partial seizures and normal MRI brain are a difficult subgroup in terms of presurgical evaluation. The diagnosis of mesial temporal lobe epilepsy in this group may be suggested by ictal semiology, interictal epileptiform discharges, or ictal EEG pattern [31]. Both ictal SPECT and interictal PET are sensitive methods for the lateralization of TLE, but ictal SPECT is more sensitive when MRI is normal [32]. Ictal SPECT and interictal PET are complementary to each other in lateralizing the side in patients with TLE and normal MRI (Figure 3).

### 2.9. SPECT in Extratemporal Lobe Epilepsy

**Figure 4:**
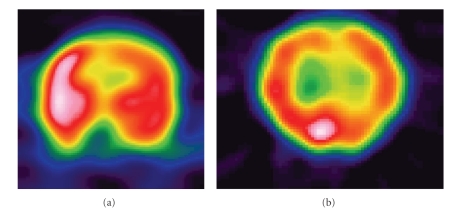
Ictal SPECT in extra temporal epilepsy showing (a) right frontal hyperperfusion in a patient with frontal lobe epilepsy (b) right occipital hyperperfusion in a patient with occipital lobe epilepsy.

In extra-temporal lobe epilepsies with normal MRI, the localization of ictal onset zone is difficult, and an extensive invasive monitoring using intracerebral grid and depth electrodes is required. In spite of invasive monitoring the outcomes in extra-temporal lobe epilepsy surgery are not as good as those achieved in temporal lobe surgery. Ictal SPECT and FDGPET will guide the placement of these electrodes. Lee et al. have shown that seizure-free outcome could be achieved in 47% and upto 90% seizure reduction could be achieved in 80% of the patients with refractory epilepsy and normal MRI, evaluated with ictal SPECT and FDGPET [33]. Ictal SPECT studies may show focal hyperperfusion and help in differentiating temporal from extratemporal epilepsy, confirm the epileptogenecity of a structural lesion, and guide the placement of intracranial electrodes in patients with normal MRI [12, 23, 34, 35]. Ictal SPECT has been demonstrated in the frontal lobes, frequently accompanied by ipsilateral basal ganglia and contralateral cerebellar hyperperfusion [34]. In parietal lobe epilepsy, anterior parietal hyperperfusion was noted with sensorimotor features and posterior parietal hyperperfusion when seizures are psychoparetic in type [36]. Very early ictal injection is required to find a focus in occipital lobe epilepsy (Figure 4). In a study of 17 patients with occipital lobe epilepsy ictal SPECT showed focal occipital hyperperfusion in only 29% [37]. It has been estimated that extratemporal seizures should last 10–15 s after ictal SPECT injection to give good localizing information [38]. Pitfalls of ictal SPECT are that ictal SPECT may show propagated ictal activity and ictal SPECT hyperperfusion does not exclude multifocal seizure onset.

## 3. SISCOM

Subtraction ictal SPECT with co-registration on MRI (SISCOM) gives good anatomic correlation and highlights an area of relative hyperperfusion or hypoperfusion not readily apparent on visual inspection. Statistical parametric mapping (SPM) improves subtraction image quality. O'Brien et al. [39] reported an excellent outcome when SISCOM localization was concordant with surgical-resection site, but not when SISCOM and resection site were discordant in patients with refractory partial epilepsy and normal MRI. In patients with normal MRI and refractory epilepsy, SISCOM may help to detect subtle focal cortical dysplasia [40]. The indications for SISCOM in patients undergoing a presurgical evaluation include nonsubstrate-directed partial epilepsy multilobar pathology and when there are conflicting results in the noninvasive evaluation [41]. The presence of a SISCOM alteration may obviate the need for intracranial EEG recordings in selected patients. Patients with refractory temporal lobe epilepsy and normal MRI may not require chronic intracranial EEG monitoring if the extracranial ictal EEG pattern and ictal SPECT studies are concordant.

### 3.1. Ictal SPECT in Other Seizure Disorders

Ictal SPECT has been used to investigate infants with infantile spasms (West syndrome). Focal cortical hyperperfusion has been shown in one-third of the cases [42]. The yield for localization in Lennox-Gastaut syndrome is very low and hence ictal SPECT has limited role in this group of patients. Ictal SPECT shows focal or regional hyperperfusion while interictal SPECT will show hypoperfusion in patients with Rassmussen's encephalitis. This may be useful in defining the site for biopsy to confirm the diagnosis [43]. Ictal SPECT shows hyperperfusion of the hamartoma in patients with hypothalamic hamartoma or may show propagation to cortical areas [44]. Ictal SPECT also helps to differentiate true from pseudoseizures when the ictal EEG does not give enough information [45].

### 3.2. Comparision of Ictal SPECT and FDGPET

In patients with temporal lobe epilepsy, ictal SPECT was found to be marginally more sensitive than FDGPET for the lateralization of the epileptogenic focus, 89% versus 83% [32]. In patients with neocortical epilepsy FDGPET was found to be more sensitive than ictal SPECT and MRI, with a sensitivity of 78%, 70%, and 60%, respectively [46]. In another study FDGPET and ictal SPECT were found to have the same sensitivity of 56% but were complementary to each other [35]. In summary interictal FDGPET and ictal SPECT have similar sensitivity to localize the seizure focus, but complementary when the other modality is not localizing in a given patient [47]. Ictal SPECT should always be read in the context of other presurgical investigations and is useful to localize the epileptogenic zone noninvasively [48].

## 4. Conclusion

Ictal SPECT is a valuable noninvasive modality during the presurgical evaluation of patients with refractory partial epilepsy. It may obviate the need for intracranial monitoring in patients with refractory temporal lobe epilepsy and normal MRI. Ictal SPECT (SISCOM) guides the placement of depth and grid electrodes in patients with refractory partial epilepsy and normal MRI. Ictal SPECT and FDGPET are complementary for localization of the seizure focus.
